# Multiple imputation strategies for missing event times in a multi-state model analysis

**DOI:** 10.1002/sim.10011

**Published:** 2024-01-22

**Authors:** Elinor Curnow, Rachael A. Hughes, Kate Birnie, Kate Tilling, Michael J. Crowther

**Affiliations:** 1 Department of Statistics and Clinical Research, NHS Blood and Transplant, Bristol, UK; 2 Department of Population Health Sciences, Bristol Medical School, University of Bristol, Bristol, UK; 3 Medical Research Council Integrative Epidemiology Unit, University of Bristol, Bristol, UK; 4 Department of Medical Epidemiology and Biostatistics, Karolinska Institutet, Stockholm, Sweden; 5 Red Door Analytics, Stockholm, Sweden

**Keywords:** Markov, missing data, multiple imputation, multi-state model, predictive mean matching

## Abstract

In clinical studies, multi-state model (MSM) analysis is often used to describe the sequence of events that patients experience, enabling better understanding of disease progression. A complicating factor in many MSM studies is that the exact event times may not be known. Motivated by a real dataset of patients who received stem cell transplants, we considered the setting in which some event times were exactly observed and some were missing. In our setting, there was little information about the time intervals in which the missing event times occurred and missingness depended on the event type, given the analysis model covariates. These additional challenges limited the usefulness of some missing data methods (maximum likelihood, complete case analysis, and inverse probability weighting). We show that multiple imputation (MI) of event times can perform well in this setting. MI is a flexible method that can be used with any complete data analysis model. Through an extensive simulation study, we show that MI by predictive mean matching (PMM), in which sampling is from a set of observed times without reliance on a specific parametric distribution, has little bias when event times are missing at random, conditional on the observed data. Applying PMM separately for each sub-group of patients with a different pathway through the MSM tends to further reduce bias and improve precision. We recommend MI using PMM methods when performing MSM analysis with Markov models and partially observed event times.

## 1 Introduction

In clinical studies, there is often interest in describing the sequence of events that each patient experiences, to enable better understanding of disease progression. Increasingly, multi-state model (MSM) analysis is used for this purpose. In the MSM framework, experiencing an event can be thought of as a move (“transition”) from one “state” to another. MSMs have been used in a wide variety of clinical contexts, such as organ and stem cell transplantation,^1,2^ studies of dementia^3^ and aging,^4^ and in cancer research.^5^ The advantage of the MSM approach is that the probability of multiple events can be modeled simultaneously. This allows the prediction of clinically-relevant quantities, such as the probability of each event at any given time, and the average number of days spent in each state. This in turn enables more effective communication of risk to patients,^6^ particularly because these quantities can easily be illustrated graphically.

A complicating factor in MSM studies is that the exact time of each event may not be known. In some settings, none of the event times are exactly observed (with the possible exception of time of death). For example, in HIV^7^ or dentistry,^8^ changes in the health of the patient are reported only at intermittent clinic visits. Formally, such events are “interval-censored”: the event time lies in the interval (*L, R*], where *L* represents the last known event-free time and *R* represents the first time at which the event is reported. In such settings, maximum likelihood (ML) methods for interval-censored data^1,3,9^—in which the marginal likelihood of the observed data is maximized—are generally used. In other settings, exact event times are observed for some individuals but not others. For example, in a pregnancy study,^10^ gestational age at delivery was recorded for some individuals but missing for others. In this type of setting, a wider choice of methods for missing data is available because some individuals have complete data. As well as ML, available methods include complete case analysis (CCA), inverse probability weighting (IPW), and multiple imputation (MI).^11^

Our motivating example is in this type of setting. We consider a previously analysed dataset of patients who received hematopoietic stem cell (HSC) transplants using cord blood (CB) donated to the UK National Health Service (NHS) Cord Blood Bank (CBB).^12^ There were missing data in the NHS CBB dataset. In particular, the times of onset of acute graft-vs-host disease^13^ (aGvHD, caused by an immune response of donor cells—the “graft”—against the patient’s tissues and organs—the “host”) and relapse (ie, signs and symptoms that the patient’s original blood disease has returned after treatment) were missing for approximately 25% of patients who experienced aGvHD and/or relapse, respectively. Note that these missing times can still be considered interval-censored, with finite interval boundaries inferred from clinical criteria (eg, the standard clinical definition of aGvHD^13^ assumes occurrence between day 0 and 100 post-transplant) or the known length of the monitoring period for each patient (exact times of death or last follow-up were reported for all patients).

The NHS CBB dataset is an interesting test case because, although possible in principle, ML, CCA, and IPW have limited use for handling missing event times, for the following reasons:

1. Our setting deviates from the assumptions of the ML methods that are, to our knowledge, available, in two ways:
  (i) Our event times are a mixture of observed and missing (interval-censored) times. ML methods developed so far assume that all times are interval-censored.
  (ii) For our missing times, the associated interval boundaries are wide relative to the observed event times. In a review of ML methods for handling interval-censoring in MSM analysis, Machado et al.^14^ found that none of the available methods performed well when censoring intervals were wide, relative to the change in hazards.
2. CCA (in which only patients with observed values for all analysis model variables are included) will give biased estimates in our setting because missingness depends on the analysis model outcome.^15^ Note that CCA estimates would only be unbiased in this setting if the probability that event times were missing did not depend on the type of event nor the event times themselves (after conditioning on the analysis model covariates).
3. IPW (in which the complete cases are weighted by the inverse of the estimated probability of being complete) is likely to perform poorly because the types of event experienced by each patient is strongly predictive of missingness of the event times, resulting in extreme weights for some individuals.^16^ In addition, like CCA, IPW estimates generally lack precision because the incomplete cases (which contain partial information about the outcome) are discarded.^11^

In contrast to approaches (1)-(3), above, MI (assuming the missingness mechanism is ignorable and the imputation model is correctly specified^17^) utilizes all available data from both patients with fully observed event times and those with partially observed event times, using observed data for the analysis model variables plus any additional variables that are predictive of the missing event times. In addition, MI can accommodate a mixture of exactly observed and missing times, plus it allows flexibility when choosing the analysis model.

The standard MI procedure consists of three steps:

1. An imputation model (usually some form of regression model) is fitted to the observed data and missing values are replaced with draws from its predictive distribution. This is repeated multiple (M) times, to give M completed datasets.
2. The analysis model is fitted to each of the M completed datasets.
3. The M sets of results are combined using Rubin’s rules.^18^

Using the NHS CBB dataset as motivation, Curnow et al.^12^ considered MI and ML strategies for handling missing event times in a competing risks analysis. They examined the extent to which interval boundaries, the data distribution, and analysis model should be accounted for in the imputation model. Similar to Machado et al.,^14^ they found that an ML approach did not perform as well as the best MI methods, resulting in some bias and under-estimation of SE. However, MI by “type 1” predictive mean matching^19^ (PMM) resulted in least biased estimates of cumulative incidence when the ignorability assumption held and, similar to previous studies,^19,20^ was robust to model mis-specification (where mis-specification occurred because eg, the imputation model assumed a linear relationship between the event times and covariates, rather than between the log-hazard and covariates). PMM is a variation on the standard MI procedure described above, in which missing values are replaced with observed values from donors with a similar predicted mean (see Section 4 for a detailed description). Furthermore, Curnow et al. found that the standard MI procedure (using draws from a linear regression model), when stratified by aGvHD, gave results comparable with PMM. Therefore, in this paper, we focus on these two MI methods, extending the work of Curnow et al. to multi-state Markov models. Note that, in this paper, we generally refer to “event” times rather than “transition” times. This is because it is more realistic to have missing times for a specific event—which may affect several transition times—rather than for a specific transition (eg, a missing time of aGvHD will affect times of transition to and from aGvHD).

In Section 2 we describe MSM methodology in detail. In Section 3 we describe the motivating example. In Section 4 we describe the MI methods in detail. In Section 5 we describe a simulation study comparing MI methods and present its results in Section 6. In Section 7 we apply our MI methods to the motivating study dataset. We conclude with general discussion in Section 8.

## 2 Multi-State Models

Formally, we consider a stochastic process up to time *τ*: {*Y*(*t*), *t* ∈ *T* = [0, *τ*]} where *Y*(*t*) denotes the state occupied by an individual at time *t*, with a finite state space *Z* = {0, …, *N*} and process history up to time *s, H*_*s*_ = {*Y*(*u*); 0 ≤ *u* ≤ *s*}.^2^ The set of transition intensities, *α*_*ab*_(*t*), defined as the instantaneous probability of moving from state *a* to state *b* at time *t*(analogous to the hazard rate in standard survival analysis), fully characterizes the multi-state process. *P*(*Y*(*t*) = *b* | *Y*(*s*) = *a, H*_*s*_) represents the transition probability, that is, the probability that a patient in state *a* at time *s* moves to state *b* at time *t*, given the process history up to time *s*, for *a, b* ∈ *Z* and *s, t* ∈ *T*, with *s* ≤ *t*.

The calculation of transition intensities and transition probabilities is most straight-forward for MSMs with the Markov property.^21^ This property states that the transition probability depends only on the current state occupied but not the amount of time spent in the current state nor the past history prior to entry into the current state. Hence, in this case, the transition probability can be simplified to *P*(*Y*(*t*) = *b* | *Y*(*s*) = *a*), hereafter denoted by *P*_*ab*_(*s, t*). Then the matrix of transition probabilities, **P**(*s, t*) = {*P*_*ab*_(*s, t*)}, can be calculated as follows (using product integral notation): **P**(*s, t*) = Π_(*s*,*t*]_ (**I** + d***A***(*u*)) where **I** is the identity matrix and ***A***(*u*) = {*A*_*ab*_(*u*)} is the matrix of cumulative transition intensities, defined as: $A_{ab}\left(u\right)=\int_{0}^{u}\alpha_{ab}\left(v\right)dv$, with α_*aa*_(*v*) = −∑_*b*≠*a*_ α_*ab*_(*v*).^2^ Note that this paper only considers Markov models. We include a test for the Markov property in the simulation and real data analysis.

## 3 Motivating Study

The NHS CBB dataset contains information about 432 CB transplants. Individual-level data are available about baseline patient, donor, and transplant characteristics (see Supplementary Material Section S4 for further details) as well as about events experienced by each patient during the post-transplant monitoring period. Event types include aGvHD and chronic graft-vs-host disease (cGvHD, occurring more than 100 days post-transplant), myeloid engraftment (defined as absolute neutrophil count >0.5 × 10^9^/L on three consecutive days), relapse, and death. The median follow-up time is 3 years (Kaplan-Meier estimate, censoring follow-up time at death) and at least one post-transplant event is reported for each patient. For each type of event, both an indicator of whether the event was experienced and the associated time of onset are reported (censoring at the earliest of the time of a competing event or last follow-up).

**Figure 1:**
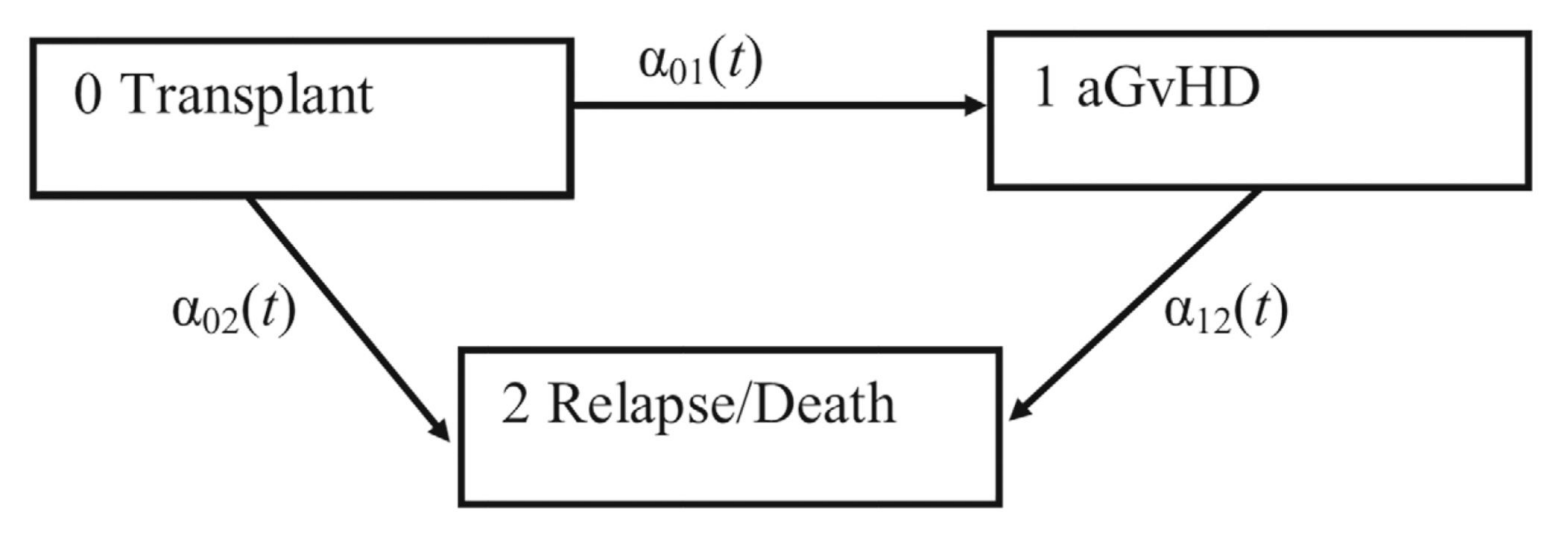
The illness-death multi-state model.

In our study, we use a MSM, as depicted in Figure 1, to explore the association between patient, donor, and transplant characteristics and the timing and probability of aGvHD, relapse, and death. In Figure 1, states are represented by rectangles, and possible transitions by arrows. The transition intensity, *α*_*ab*_(*t*), defined above, is shown for each transition. There is a single initial state (0 Transplant), and we assume that all patients are in this state at the time origin, *t* = 0. There is a single “absorbing” state (2 Relapse/Death), that is, a state from which further transitions cannot occur (this composite outcome, defined as the earliest of either relapse or death, is of clinical interest in many HSC transplant studies^22–24^; we note that in some situations, patients may receive further treatment and recover from relapse^25^ but here we do not consider transitions to/from relapse separately from those to death). There is also an “intermediate” state (1 aGvHD) between initial and absorbing states. There are two possible pathways through this MSM: either 0-1-2 (patient *i* is transplanted at *t* = 0, experiences aGvHD at time *t*_1*i*_, and experiences relapse or death at time *t*_2*i*_, with 0 < *t*_1*i*_ < *t*_2*i*_), or 0-2 (patient *j* is transplanted at *t* = 0, and has not experienced aGvHD prior to relapse or death at time *t*_2*j*_, with *t*_2*j*_ > 0). As per standard survival analysis, patients can be right-censored at any time-point along their pathway. More complex MSMs may have multiple initial, intermediate, or absorbing states, and bi-directional arrows, but these types of MSM are out of scope for this paper.

As described in the Introduction, the challenge with our study is that the times of aGvHD and/or relapse are missing for some patients. The missing data patterns of the times of aGvHD and relapse, according to the combination of events experienced, are summarized in Table 1. Table 1 shows that 69 (16%) patients are missing the time of either aGvHD or relapse, with a further 5 (1%) patients missing both times. Note that for patients who experienced relapse but are missing the associated time, this implies that the time of the composite event “relapse/death” is also missing. In general, calculation of transition times and related estimands (eg, transition intensities) requires the time of entry into both the starting state and terminating state to be observed.^26^ Hence, for patients with missing times of aGvHD and/or relapse/death, the associated times of any transitions to or from these states are also missing.

## 4 Multiple Imputation Methods

The MI methods we consider are based on either PMM, or MI using draws from a linear regression model. Note that these are the default imputation methods for continuous variables in several software implementations of MI (PMM is the default when using *mice* in R^27^ and MI using draws from a linear regression model is the default when using *mi impute* in Stata^28^ or *proc mi* in SAS^29^).

The general approach for these two methods is as follows.

### 4.1 PMM

In PMM, for each patient *i* with a missing value for variable *X*, the following steps are performed:

1. Calculate the predictive distance for all *h* subjects with an observed value for *X*. We use “type 1” PMM, in which the predictive distance is calculated as $\left|{\left(\theta^{*}\right)}^{T}w_{i}-{\left(\hat{\theta }\right)}^{T}w_{h}\right|,$, where (.)^***T***^ denotes the transpose function, $\hat{\theta }$ denotes the estimates of regression coefficients ***θ*** from a regression of *X* on predictors **W**, fitted to all *h* observed values of *X*; θ* denotes a random draw from the posterior distribution of ***θ***; and ***w***_***i***_ and ***w***_***h***_ denote the values of **W** for subjects *i* and *h*, respectively.
2. Identify a donor pool of subjects for which the predictive distance is minimized
3. Randomly select a subject *d* from the donor pool
4. Replace the missing value of subject *i* with the observed value of subject *d*.

### 4.2 MI using draws from a linear regression model

In MI using draws from a linear regression model, missing values of *X* are drawn from its posterior predictive distribution, conditional on regression coefficients θ* and predictors **W**, with θ* and **W** defined as above.

### 4.3 MI methods used in the simulation study

We considered four variations on the MI approaches described above to accommodate particular features of the data, as follows:

1. PMM, fitting a single imputation model for all patients.
2. PMM, applying separate imputation models for patients who did and did not experience aGvHD before relapse/death (PMMSUBGP).
3. MI using draws from a linear regression model, applying separate imputation models for patients who did and did not experience aGvHD before relapse/death (LINMI). Any negative imputed times were replaced by the value 0.0001 post-imputation.
4. PMM, compatible with the ordered nature of the event times as specified in the analysis model (PMMCOMP). In this method, PMM is applied using separate imputation models for patients who did and did not experience aGvHD before relapse/death. For patients who experienced aGvHD, the (calculated) time from aGvHD to relapse/death is used in the imputation procedure, instead of the time from transplant to relapse/death. This method proceeds as follows:
  (i) For the first event experienced (ie, relapse/death for the subgroup of patients who did not experience aGvHD, and aGvHD for the subgroup who did), impute any missing event times using PMM.
  (ii) For the subgroup of patients who experienced aGvHD, also impute any missing times from aGvHD to relapse/death using PMM. Post-imputation, calculate any missing times to relapse/death as the sum of the (observed or imputed) time to aGvHD and the (observed or imputed) time from aGvHD to relapse/death.

Methods (2)-(4) use the stratified approach found by Curnow et al.^12^ to improve bias and precision of the MI estimates, allowing for different distributions of event times for those who experience aGvHD compared with those who do not experience aGvHD before relapse/death. In addition, method (4) explicitly accounts for the ordered nature of the event times.

### 4.4 Choice of predictors

Following current guidelines,^30^ the set of predictors **W** consisted of all other analysis model variables, that is, all analysis model covariates, the time of the other event, and an indicator of relapse/death (with a value of 1 indicating relapse/death was experienced, and 0 otherwise). For method (1), PMM, which did not involve stratifying by aGvHD, **W** also included an indicator of aGvHD. We assumed a linear relationship between the imputed variable and its predictors, with no interactions. For example, using PMM with two covariates, *z*_1_ and *z*_2_ (further defined below), the linear predictor, ***θ***^***T***^**W**, is as follows, when imputing the time of aGvHD:

$$\theta_{0}+\theta_{1}z_{1}+\theta_{2}z_{2}+\theta_{3}\times \text{time of relapse/death }+\theta_{4}\times \text{indicator of relapse}/\text{death}+\theta_{5}\times \text{indicator of aGvHD}\;$$

and is as follows, when imputing the time of relapse/death:

$$\theta_{0}+\theta_{1}z_{1}+\theta_{2}z_{2}+\theta_{3}\times \text{time of aGvHD}+\theta_{4}\times \text{indicator of relapse/death}+\theta_{5}\times \text{indicator of aGvHD}\;$$

Note that we did not treat censored times as missing that is, a patient who was still alive without relapse would have an event indicator for relapse/death equal to 0, with an associated event time equal to the latest follow-up time; similarly, a patient who experienced relapse/death without aGvHD would have an event indicator for aGvHD equal to 0, with an associated event time equal to the time of relapse/death.

### 4.5 Multiple imputation with more than one partially observed variable

In settings in which times of both aGvHD and relapse/death were partially observed, the above methods were applied using the fully conditional specification (FCS, also known as “chained equations”) multivariate MI approach.^31^ This involves imputing the time of aGvHD and the time of relapse/death using separate models (although using the same MI approach in both models ie, both PMM, both PMMSUBGP, etc.), with each model sampled from in turn, conditional on all other observed and imputed data, in an iterative process. Thus, as described above, the set of predictors, **W**, for the imputation model for time of aGvHD includes the (observed or imputed) time of relapse/death; similarly, for time of relapse/death, it includes the (observed or imputed) time of aGvHD. *C* iterations are performed and values from the *C*^th^ iteration are retained as the imputed dataset. The process is repeated *M* times to create *M* imputed datasets.

## 5 Simulation Study

We conducted a simulation study to assess the performance of the MI methods described above, when applied to a MSM analysis, when some event times were missing. The aim of the simulation study was to compare the bias and precision of estimates from a MSM analysis when using different MI strategies, in various missing data scenarios. The design of the simulation study is summarized below.

### 5.1 Data generation

We first generated complete data for the event times and associated states, using the MSM set-up depicted in Figure 1, using the method described by Beyersmann et al.^32^ (see Supplementary Material Section S1 for further details) under the following assumptions:

- The time of transplant was known, that is, the time origin was observed for all patients.
- Subsequent events could be unobserved, that is, there was right-censoring. The censoring distribution was assumed to be independent of the event time and state occupied.
- For patients who experienced both aGvHD and relapse/death, we assumed that aGvHD always occurred before relapse/death.
- All transitions had the Markov property.
- Each transition intensity model had a proportional hazards (PH) structure. This meant that the transition intensity, *α*_*ab*_(*t*), at time *t* since transplant, when moving from state *a* to state *b*, was defined for each patient *i* with time-fixed covariates ***z***_***i***_ as follows: $\alpha_{ab}(t)=\alpha_{ab}^{0}(t)\;\text{exp}\left(\beta_{ab}^{T}z_{i}\right)$, where $\alpha_{ab}^{0}(t)$ represents the baseline intensity at time *t*. We assumed a Weibull distribution for each baseline intensity.

Specifically, data were generated based on the following transition intensity models (for transitions 01: transplant to aGvHD, 02: transplant to relapse/death, 03: aGvHD to relapse/death):

$$\begin{array}{c} \alpha_{01}(t)=\left(\frac{1.5}{36}\right){\left(\frac{t}{36}\right)}^{0.5}\text{exp}\left\{-0.8z_{1}\right\}.\\ \alpha_{02}(t)=\left(\frac{0.9}{120}\right){\left(\frac{t}{120}\right)}^{-0.1}\text{exp}\left\{1.2z_{1}\right\}.\\ \alpha_{12}(t)=\left(\frac{0.8}{160}\right){\left(\frac{t}{160}\right)}^{-0.2}\text{exp}\left\{1.2z_{1}-z_{2}\right\}. \end{array}$$

where *t* represents the time in days since transplant, *z*_1_ ~ *Bernoulli*(0.2) represents whether a patient is in relapse at time of transplant (assuming patients in relapse at the time of transplant are relapse-free immediately post-transplant), *z*_2_ ~ *Bernoulli*(0.45) represents whether a patient receives a double cord transplant (vs single cord), and *z*_1_ and *z*_2_ are independent. The magnitude of the model parameters and choice of covariates were based on the real data. Censoring times were randomly generated between one and 5 years post-transplant, to represent administrative (non-informative) censoring at study end. We used 1000 simulations and each simulated dataset contained 500 patients (similar to the size of the real dataset).

In a subsequent step, missing event times were generated using 12 different missing data mechanisms (MDMs). First, we considered event times missing completely at random (MCAR, ie, missingness was independent of both the observed and missing data) by setting a random 30% of event times to missing, regardless of the event type. Next, we considered 11 different MDMs (Table 2). We assumed that event times to acute GvHD and/or relapse/death were either (a) missing at random (MAR), conditional on the observed data (missingness depended on the event type and covariates but not on the missing data itself), or (b) missing not at random (MNAR, missingness depended on the missing data itself). Although our chosen MI methods assumed data were MAR, MNAR MDMs allowed us to assess the impact on bias and precision when the MAR assumption was violated. Approximately 30% of event times were missing in each MDM, to reflect the percentage of missing times in the real data.

### 5.2 Analysis model, estimands and performance measures

Consistent with the data-generating mechanism (DGM), we fitted PH regression models for each transition intensity that is, we fitted models of the form: $\alpha_{ab}(t)=\alpha_{ab}^{0}(t)\;\text{exp}\left(\beta_{ab}^{T}z_{i}\right)$, where $\alpha_{ab}^{0}(t)$ represents the baseline intensity at time *t* when moving from state *a* to state *b*, ***β***_***ab***_ is the vector of regression parameters, and ***z***_***i***_ are the set of (time-fixed) covariates for patient *i*. As per the DGM, ***z***_***i***_ = *z*_1*i*_ for the transitions from the transplant state, and ***z***_***i***_ = (*z*_1*i*_ *z*_2*i*_) for the transition from aGvHD to relapse/death, where, as before, *z*_1*i*_ represents whether a patient is in relapse at time of transplant and *z*_2*i*_ represents whether a patient receives a double cord transplant (vs single cord).

We fitted both Cox and Weibull models. We fitted Cox models because they are commonly used in practice. In case of bias due to mis-specification of the baseline intensity function (because it is estimated non-parametrically in the Cox model), we also fitted Weibull PH models (ie, using the same form for the baseline intensity as the DGM).

In our analysis, the estimands of interest were:

1. The vector of transition intensity regression parameters ***β***_***ab***_ for all possible states *a* and *b*.
2. The restricted expected length of stay (RELOS) in each state,^4^ restricted to the time period between transplant and 2 years post-transplant, was used as a summary of the transition probability distributions.
  RELOS from time 0 to time *t* for state *b* is defined as:
$$e_{b}\left(t\right)=\int_{0}^{t}P_{b}\left(u\right)du$$
  where the state occupation probability, *P*_*b*_(*t*), denotes the probability of being in state *b* at time *t*.^4^ If all patients are in state 0 initially, *P*_*b*_(*t*) is equivalent to the transition probability from state 0 to state *b* at time *t*, that is, *P*_*b*_(*t*)= *P*_0*b*_(0, *t*).^21^
  We calculated *e*_*b*_(*t*) using the consistent estimator^4^:
$${\hat{\text{e}}}_{b}(t)=\sum_{m=0}^{M}{\hat{\text{P}}}_{b}\left(t_{m}\right)\cdot \left(t_{m+1}-t_{m}\right)$$
  where ${\hat{P}}_{b}\left(t_{m}\right)$ is the estimated state occupation probability for state *b* at time *t* and *t*_0_ < *t*_1_ < … < *t*_*M*_ ≤ *t*_*M*+1_ are the set of ordered times from time 0 up to time *t*, across all transitions. For Cox models, the set of times was the set of all simulated times for the *k*^th^ simulation. For Weibull models, the set of times was specified as the set of all values of *t* from time 0 up to time *t*, in increments of 0.1 days.
3. The final estimand of interest, regression parameter *γ*_12_, was used to test whether our MI approach led to conclusions about the Markov assumption that were consistent with the DGM.^26^ In this test, time from transplant until aGvHD, denoted by *d*, was included as an additional covariate in the model for *α*_12_ (for the transition from aGvHD to relapse/death), that is, we fitted the model: ${\text{α}}_{12}(t)=\alpha_{12}^{0}(t)\;\text{exp}\left(\gamma_{12}d+\beta_{12}^{T}z_{i}\right)$. Since our model is Markovian, in truth, *γ*_12_ equals zero, that is, transition intensity *α*_12_ is not related to the time from transplant to aGvHD. In other settings, it may be appropriate to allow transition intensities to depend on the time(s) of entry into earlier states.

Performance measures for regression parameters ***β***_***ab***_ and RELOS were standardized bias (defined as bias/SD of the per-simulation estimates), average model-based SE, and coverage (ie, the percentage of within-simulation 95% confidence intervals that included the true value). The performance measure of interest for the regression parameter *γ*_12_ was the coverage of the 95% confidence interval. The true values of the regression parameters were as per the DGM. The true values of RELOS were calculated using numerical integration.

Model-based SE of the regression parameter estimates was calculated using standard methods.^33,34^ We calculated the model-based SE of RELOS for Cox models using a non-parametric bootstrap estimator,^35^ with 50 bootstraps per simulated dataset, and the model-based SE of RELOS for Weibull models using the delta method. As part of a separate simulation study to identify the best estimator of model-based SE of RELOS for Cox and Weibull models (see Supplementary Material, Section S2), we explored whether increasing the number of bootstraps to 500 per simulated dataset improved the performance of the bootstrap estimator. We found that although estimates of SE of RELOS were slightly smaller when using 500 rather than 50 bootstraps, any difference in coverage was negligible (with coverage close to the nominal value when the estimate of RELOS was unbiased). Therefore, we concluded that using 50 bootstraps would not unduly influence our results.

### 5.3 MI methods

We performed MI using methods (1)-(4), described in Section 4, namely PMM, PMMSUBGP, LINMI, and PMMCOMP. Methods (3) and (4) were applied in the MCAR scenario. Due to their relatively poor performance, we did not apply these methods in other scenarios. For comparison purposes, we also performed CCA because this method is often used in practice (and is the default method when there are missing values in most statistical software).

We used default settings for the number of imputations, iterations, and size of the donor pool for PMM, PMMSUBGP, and PMMCOMP (five in each case). We explored whether a larger number of imputations would change our results by also implementing PMM using 30 imputations for the MCAR scenario (referred to as PMM30IMP), noting that MI inference is still valid for a small number of imputations.^36^ We confirmed that convergence was achieved within five iterations by examining trace plots^27^ for a randomly chosen simulated dataset for each MI method and MDM. Note that, in missing data scenarios in which only one variable (the time of aGvHD or relapse/death) was incomplete, no iteration was required.

### 5.4 Computer software

Regression parameter estimates and SEs for Cox and Weibull models were calculated using “survival”^33^ and “flexsurv”^34^ R packages, respectively; state occupation probability estimates were calculated using the “mstate” R package^37^; MI methods were implemented using the “mice” R package.^27^ R code to perform the simulation study is provided in Supplementary Material Section S5.

## 6 Simulation Study Results

**Figure 2:**
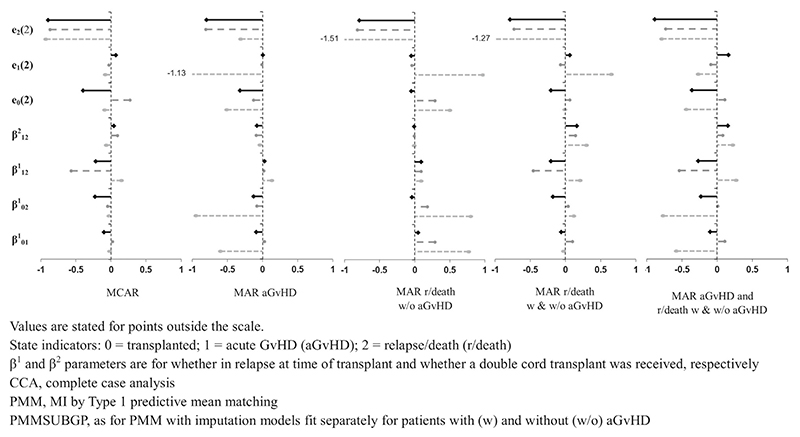
Lollipop plot of standardized bias of transition intensity regression parameters, **β**_**ab**_, and expected length of stay in each state up to 2 years post-transplant, *e*_*b*_ (2), given event times missing completely at random (MCAR) and missing at random (MAR), comparing CCA (light gray oval with dotted line), PMM (dark gray circle with dashed line), and PMMSUBGP (black diamond with solid line).

**Figure 3:**
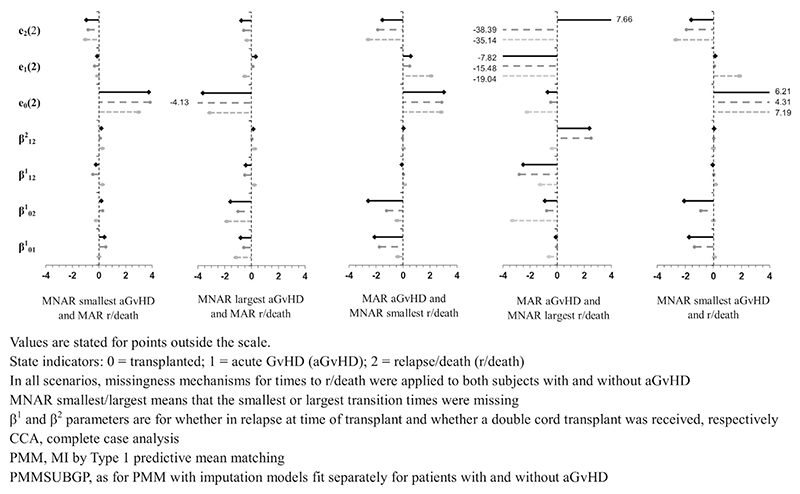
Lollipop plot of standardized bias of transition intensity regression parameters, **β**_**ab**_, and expected length of stay in each state up to 2 years post-transplant, *e*_*b*_ (2), given some event times missing not at random (MNAR), comparing CCA (light gray oval with dotted line), PMM (dark gray circle with dashed line), and PMMSUBGP (black diamond with solid line).

Simulation study results are illustrated in Figures 2 and 3 using “lollipop” plots and all results are included in the Supplementary Material Table S3a-c. Figures 2 and 3 show the standardized bias of transition intensity regression parameters **β**_ab_ for each transition, and RELOS within 2 years, *e*_*b*_ (2), for each state, fitted using a Cox model. Results are illustrated for CCA and the two main MI methods (ie, the methods that were applied in all scenarios): PMM and PMMSUBGP. Bias and model-based SE are not illustrated because these could not be shown on the same scale for all estimands, and because model-based SE was generally similar for all MI methods and MDMs (and always larger for CCA than for MI methods). Similarly, coverage rates for regression parameters and RELOS are not illustrated because these were generally similar for all MI methods, and close to the nominal value for scenarios/estimands with little bias, with under-coverage as expected where there was bias (Supplementary Material, Table S3c). Coverage of the Markov test parameter, *γ*_12_, is not illustrated because with one exception, discussed later, it was generally similar for all MI methods and MDMs.

Figure 2 shows results for scenarios in which MI was expected to work well, that is, when all event times were either MCAR or MAR (Scenarios 1, 3, 4, and 6 from Table 2 are illustrated). Conversely, Figure 3 shows results for scenarios in which MI was not expected to work well, that is, when some or all transition times were MNAR (Scenarios 7-11 from Table 2 are illustrated). For comparison, CCA estimates are also illustrated.

As expected, CCA gave unbiased estimates only when event times were MCAR. When event times were either MCAR or MAR (Figure 2), PMM resulted in a small amount of bias for all estimands (ie, the magnitude of the standardized bias was <0.5), except for *e*_2_(2) (RELOS for the relapse/death state) and the regression parameter $\beta_{12}^{1}$ (for the covariate “in relapse or not at time of transplant” in the transition intensity model from aGvHD to relapse/death). The bias in the RELOS estimate, *e*_2_(2), due to the large time intervals between individual simulated relapse/death event times (see Supplementary Material Section S2 for further details), remained for all imputation methods and MDMs when fitting a Cox model, so is not further discussed here. Bias for regression parameter $\beta_{12}^{1}$ was large in scenarios when event times to relapse/death after aGvHD were missing and small when only event times to aGvHD or relapse/death without aGvHD were missing. In the MCAR scenario, estimates of model-based SE using PMM were very similar whether using five or 30 imputations (see Supplementary Material, Table S3a,b).

Applying PMM separately for patients who did and did not experience aGvHD before relapse/death (PMMSUBGP) or accounting for the ordered nature of the event times as specified in the analysis model (PMMCOMP) reduced the bias in regression parameter $\beta_{12}^{1}$. When these methods were used, bias remained small for all other estimands except the RELOS estimate, *e*_2_(2). Model-based SE was slightly smaller for PMMSUBGP compared with PMM and larger for PMMCOMP than for other MI methods, with respect to regression parameters $\beta_{12}^{1}$ and $\beta_{12}^{2}$ (see Supplementary Material, Table S3a,b). The larger SE for PMMCOMP is because, in this method, not all available information is used about patients with a missing aGvHD time and an observed relapse/death time (for these patients, both the aGvHD time and the time from aGvHD to relapse/death are imputed, with the analysed relapse/death time calculated, post-imputation, from these imputed times). Results using PMMSUBGP were very similar for both Cox and Weibull models, except that the bias in the RELOS estimate, *e*_2_(2), was greatly reduced when fitting a Weibull model (because estimation did not rely on the individual simulated relapse/death event times as it did for Cox models). MI using draws from a linear imputation model (LINMI) resulted in large bias for some estimands, particularly estimates of RELOS (see Supplementary Material for PMMCOMP and LINMI results).

When some or all event times were MNAR (Figure 3), MI using either PMM or PMMSUBGP led to biased estimates. Bias was generally the same or larger than when using CCA. Using MI, bias was larger when the time to the absorbing state (relapse/death) was MNAR than when the time to the intermediate state (aGvHD) was MNAR, and when the largest times were MNAR than when the smallest times were MNAR. Times to relapse/death tended to be longer for patients who experienced aGvHD than for patients who experienced relapse/death without aGvHD (with aGvHD: median 200 days, IQR 386 days; without aGvHD: median 14 days, IQR 22 days). Therefore, MNAR mechanisms where longer times to relapse/death tended to be missing mainly affected patients who experienced aGvHD before relapse/death. Conversely, MNAR mechanisms where shorter times to relapse/death tended to be missing mainly affected patients who experienced relapse/death without aGvHD. This may explain why parameter estimates for the aGvHD to relapse/death transition intensity model, $\beta_{12}^{1}$ and $\beta_{12}^{2}$, were more biased than parameter estimates for the models of transition from transplant, $\beta_{01}^{1}$ and $\beta_{02}^{1}$, when the largest relapse/death times were MNAR and vice versa when the smallest relapse/death times were MNAR.

As a test of the Markov assumption, the time from transplant until aGvHD was added as a covariate to the transition intensity model from aGvHD to relapse/death. Coverage for the regression parameter for this covariate, *γ*_12_, was in the range 0.92-0.98 in all methods and scenarios, except one. The coverage was 0.66 when applying MI using the PMMSUBGP method and a Weibull analysis model, with aGvHD times MAR and largest relapse/death times MNAR. To allow further exploration of this outlying value for coverage, performance measures for the regression parameter *γ*_12_ are shown in Table 3, for all scenarios in which times to aGvHD were MAR, times to relapse/death times MNAR and the imputation method was PMMSUBGP.

As discussed above, MNAR mechanisms in which smallest times to relapse/death times tended to be missing affected mainly patients who experienced relapse/death without aGvHD. Hence, the regression parameter *γ*_12_ is unbiased with coverage close to the nominal value in this scenario. In MNAR mechanisms in which largest times to relapse/death tended to be missing, there is little bias when fitting a Cox model. However, the model-based SE is larger, which may explain the slight over-coverage in this case. Bias is large when fitting a Weibull model, which may explain the high degree of under-coverage in this case.

## 7 Analysis of the Motivating Example

To illustrate our methods, we present an analysis of the NHS CBB dataset. As per the simulation study, our interest was in estimating transition intensity model parameters and RELOS (we estimated RELOS only within 1 year because event times were sparse beyond this point). Note that our analysis model represents a very simplified version of the events experienced by patients after HSC transplantation. Hence, our results are not intended to be used for clinical insight.

### 7.1 Methods

#### 7.1.1 Analysis model

We fitted the three-state Markov model used in the simulation study, using a PH regression model for each transition intensity (fitting Cox models for all missing data methods, and additionally fitting Weibull models for PMMSUBGP). Transition intensity models included all clinically relevant baseline (at time of transplant) covariates. We tested the analysis model assumptions as follows:

1. The PH assumption was tested for each transition intensity model using the global test (ie, testing for proportional hazards across all covariates in combination) proposed by Grambsch and Therneau.^38^
2. As a test of the Markov assumption, an additional model was fitted for the transition from aGvHD to relapse/death, including the time from transplant until acute GvHD as well as all covariates.

#### 7.1.2 Missing data methods

In the NHS CBB dataset, both event times and some covariates were partially observed (see Supplementary Material, Section S4). For simplicity, and illustration purposes only, we assumed all data were MAR (see Curnow et al.^12^ for discussion of potential missingness mechanisms for this dataset). Therefore, we applied FCS MI (using the “mice” R package, as before). Covariate data were imputed using standard methods: binary variables using logistic regression, and categorical variables using multinomial regression models. Missing event times were imputed using the main MI methods used in the simulation study (ie, PMM and PMMSUBGP). Here, the sub-groups used in the PMMSUBGP method were:

1. Patients experiencing both aGvHD and cGvHD, or cGvHD without aGvHD (N = 82).
2. Patients experiencing aGvHD without cGvHD (N = 173).
3. Patients experiencing relapse without GvHD, or neither relapse nor GvHD (N = 177).

For each MI method, the imputation model for each partially observed variable included all other analysis variables, that is, all other covariates, event indicators, and event times. Year and country of transplant, and whether the patient experienced cGvHD and/or myeloid engraftment, and the associated event times, were also included in each imputation model as auxiliary variables because they were highly predictive of both missingness and the incomplete variables themselves. Note that indicators of whether the patient experienced aGvHD or cGvHD (and for group (3), the indicator of relapse/death) were excluded from each imputation model when using the PMMSUBGP method because these had the same value for all patients in each sub-group. The time of the composite event (relapse/death) was derived post-imputation.

It is well-established when imputing covariates in a survival analysis that, to ensure compatibility (or approximate compatibility) with the analysis model, both event indicators (binary variables indicating whether each event was experienced) and a representation of the distribution of the associated event times should be included in the imputation model for each partially observed covariate.^39–41^ Since both covariate data and event times were missing in the NHS CBB dataset, the actual event times were included in the imputation models, rather than, for example, the baseline hazard function recommended by White and Royston.^40^ We performed 80 imputations (following the “rule of thumb”^42^ that the number of imputations should at least equal the percentage of incomplete cases—73% in the NHS CBB dataset). As in the simulation study, we used the default of five iterations per imputation (assessing convergence using trace plots as before) and a donor pool of five donors for each PMM method. We also calculated CCA estimates for comparison purposes. We used 500 bootstrap samples in estimation of the SE of RELOS for Cox models, using a non-parametric bootstrap estimator as per the simulation study (increasing the number of bootstraps compared with the simulation study because the real data may not be as well-behaved).

### 7.2 Results

**Figure 4:**
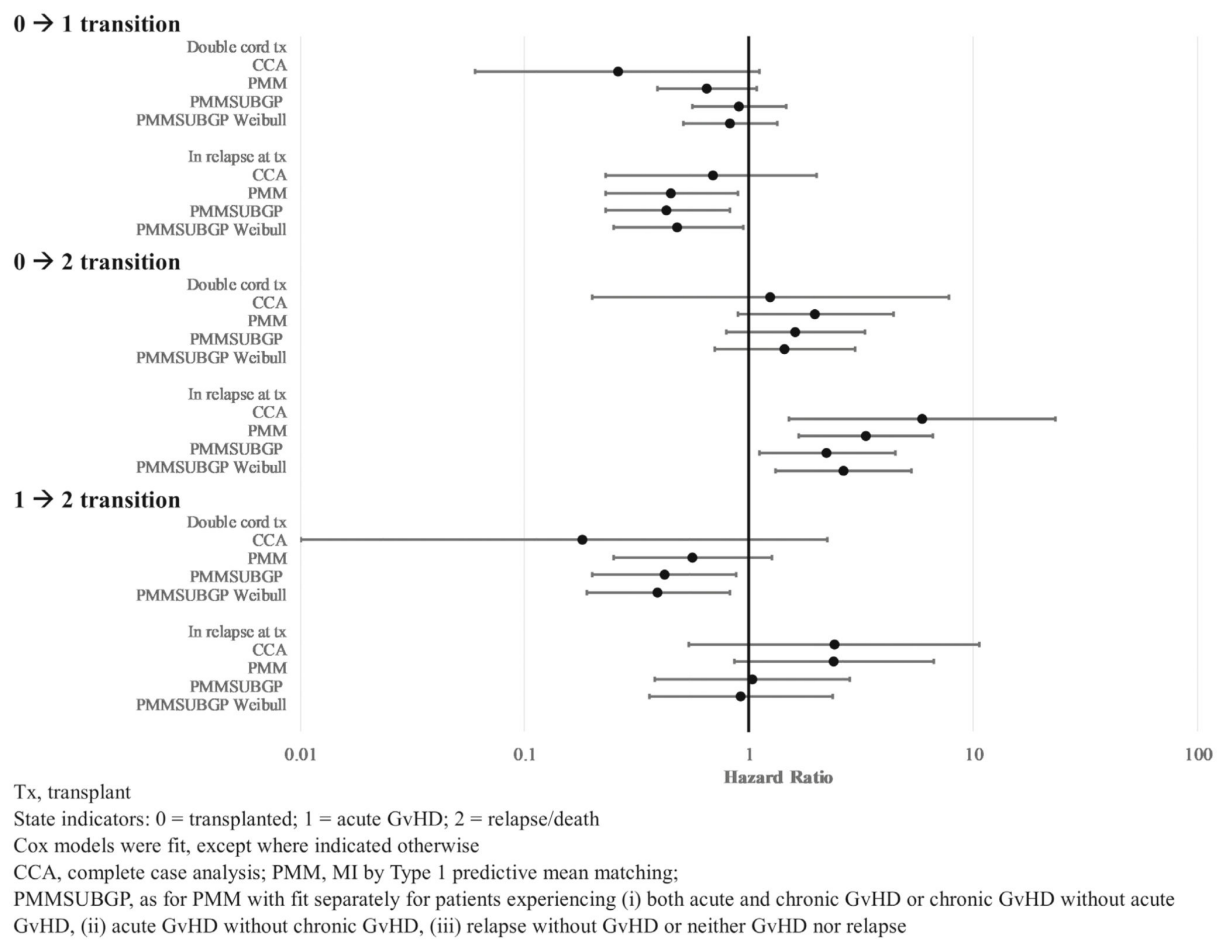
Hazard ratio estimates and 95% confidence intervals for each transition, comparing CCA and MI methods.

To illustrate the difference between estimates from CCA and MI methods (PMM and PMMSUBGP, fitting either a Cox or Weibull model for the latter method), Figure 4 shows estimated hazard ratios (HR, conditional on all other covariates) for a double cord transplant (vs single cord) and whether a patient was in relapse at time of transplant (vs in remission) for each transition (see Supplementary Material Table S4a-c for full results). For each HR, 95% confidence intervals (CI) were wider for CCA estimates than for MI estimates. PMMSUBGP estimates were very similar, whether a Cox or Weibull method was fitted. PMM estimates were generally similar to PMMSUBGP estimates. Some CCA point estimates were outside the 95% CI for the equivalent MI estimates (this was the case for double cord transplant, for both the transition from transplant to aGvHD and the transition from aGvHD to relapse/death, and, for some MI estimates, for whether in relapse at time of transplant, for both the transition from transplant to relapse/death and the transition from aGvHD to relapse/death). In these cases, the MI estimates were closer to the null than the CCA estimate.

Table 4 shows CCA and MI estimates of RELOS in the first year post-transplant (illustrated for three different patient types: a patient with reference values of covariates, a low-risk, and a high-risk patient). For each patient type, CCA estimates of the time spent in relapse/death were higher than the MI estimates whilst CCA estimates of time spent in the aGvHD state were lower. For all estimates, CIs were wide and generally widest for CCA estimates (although in some cases, particularly for estimates for a high-risk patient, CIs of CCA estimates were narrower than CIs of MI estimates due to truncation of intervals outside the plausible range [0, 365]). CIs were also wide for the aGvHD and relapse/death states when fitting a Weibull model (PMMSUBGP Weibull). This may be due to the small number of transitions relative to the range of observed event times for example, only 46 patients with reference values of covariates experienced relapse/death after acute GvHD, and the range of event times was 14-1711 days post-transplant. As a consequence, convergence of the Weibull model was not achieved in 29 of the 80 imputed datasets for the transition from aGvHD to relapse/death.

There was no apparent association between time of acute GvHD and the hazard of relapse/death after acute GvHD (HR 1.00, 95% CI 0.99-1.01, see Supplementary Material, Table S4d), suggesting there was no violation of the Markov assumption. There was some indication of a violation of the PH assumption, particularly for the model for the transition from transplant to relapse/death (global PH test *P*-value = 0.14, 0.03, 0.30 for the transitions from transplant to acute GvHD, transplant to relapse/death and acute GvHD to relapse/death, respectively).

## 8 Discussion

In this paper, by simulation, we have shown that MSM analysis using Markov models with an MI strategy based on PMM yields estimates with little or negligible bias when event times are MAR. In our setting, in which the probability that event times are missing depends on the event type (a common occurrence in practice because overall survival status is generally completely reported whereas non-fatal events may not be), CCA is not valid because missingness depends on the analysis outcome. Our simulation study shows that even when CCA estimates are unbiased (eg, when times are MCAR), PMM estimates have better precision than CCA estimates. In PMM, missing values are replaced by sampling at random from a donor pool of patients with observed values who are ‘similar’ to the subject with missing data. In the MSM context, this means that the donor pool tends to contain patients who have experienced the same sequence of events as the incomplete case. Therefore, the original sequence of events can be preserved for the incomplete case, without explicitly specifying the order of events in the imputation process. In both our simulation study and real data application, the distribution of event times differed across sub-groups of patients. In the simulation study, applying PMM separately for sub-groups of patients who did and did not experience the intermediate event (PMMSUBGP) tended to reduce bias and model-based SE, particularly for parameters for the transition from the intermediate to absorbing state (aGvHD to relapse/death). PMMSUBGP also improved coverage in a parameter used to test the Markov assumption (by including time from transplant to aGvHD in the model).

An extension of the PMMSUBGP method, which explicitly preserved the ordering of events by including the aGvHD event time and time from aGvHD to relapse/death in the imputation model, but not the relapse/death event time (PMM-COMP), gave results with comparable bias to PMMSUBGP, but larger model-based SE. Due to the loss of information using this method, with no advantage in terms of bias reduction, we would not recommend this approach.

In our study, MI using draws from a linear imputation model (LINMI) led to more bias than PMM when estimating transition intensity model parameters and RELOS. This may be because this approach could result in an imputed relapse/death time that was smaller than the (observed or imputed) aGvHD time. Hence, LINMI was not compatible with the analysis model and estimates were biased as a consequence.

Overall, we recommend using Type 1 PMM to impute missing event times in a MSM analysis using Markov models, first exploring the distribution of event times for each sub-group of patients with a different path through the MSM. Type 1 PMM should be applied separately for each sub-group of patients with a different distribution of event times. In our simulation study, the distributions of simulated times of relapse/death were very different for patients who did and did not experience aGvHD. In analysis of real data, there may be smaller differences between distributions of event times for different sub-groups of patients, and applying PMM by sub-group may make little difference to the results. Therefore, to assess the sensitivity of results to the imputation method, we recommend performing analysis using both a single imputation model and separate models for each sub-group of patients. Note that sub-groups should be of sufficient size to allow for random donor selection in the PMM procedure.

The PMM strategy described here can only be used if some event times are exactly observed, which may not always be the case. For example, after corneal transplantation, hospitals were asked whether any post-transplant surgery had been performed since the previous follow-up report but were not asked for the date of surgery.^43^ In this example, time of surgery would be missing for all patients. Valid use of PMM would require further data collection to obtain exact event times for a representative sample of patients. If this was not possible, a ML approach could be used instead.

Although PMM performed well in our study, there is still scope for improvement, for example, by development of methods that are explicitly compatible with a MSM analysis. This could be achieved, for example, through an extension of the MAR stacked MI approach of Beesley and Taylor^44^ or the SMC-FCS method^45^ to MSM, particularly when these use parametric models. Alternatively, another method proposed by Beesley and Taylor^46^ could be extended, combining an ML approach with full imputation (Beesley and Taylor use “improper” imputation within their EM algorithm). We note that the purpose of Beesley and Taylor’s approach was primarily to impute missing event states. This is different from our set-up, in which we assume that all event states are known.

Generally, MI techniques that assume MAR are not recommended when data are MNAR. In this study, MI resulted in biased estimates when event times were MNAR or a mixture of MAR and MNAR. Our results suggested that bias was greater when the time to the absorbing state (relapse/death) was MNAR than when the time to the intermediate state (aGvHD) was MNAR. This may be due to the constrained nature of the time to an intermediate event (in an illness-death model, this is bounded by 0 and the time of transition to the absorbing state), which may limit the degree of bias even when event times are MNAR. Conversely, the lack of constraint on the maximum time of transition to an absorbing state, and the different pathways through the MSM to that state (each potentially with a different distribution of event times), may increase the degree of bias. Here, we only considered MSMs with the Markov property. Semi-Markov or non-Markov models may result in greater bias when intermediate state event times are MNAR. Therefore, further work is needed to determine if the conclusions of this research still hold for more complex MSMs. A further, useful, extension of this research would be to consider a range of sample sizes, covariate associations, and event rates.

In our simulation study, parametric analysis models generally performed as well as semi-parametric models. Furthermore, parametric models resulted in less biased estimates of the expected length of stay in state (RELOS) when there were sparse event times. However, regression parameter estimates from parametric models were more biased than estimates from semi-parametric models when event times were MNAR. In addition, in practice, parametric models seemed to be more prone to convergence problems than semi-parametric models (and this may have been the case even if all variables were fully observed because event times were sparsely distributed). Further work is required to determine if this is also the case for flexible parametric models.

In the real data analysis, there was some indication that the proportional hazards assumption did not hold, particularly for the model for the transition from transplant to relapse/death. Therefore, the model could be improved by including time-dependent regression parameters, or by using the dynamic landmarking approach.^47^ In addition, clinical inference would be strengthened, and important clinical questions could be answered, if a more detailed event history was modeled for each patient. However, this does rely on the availability of additional post-transplant data, which will almost certainly include some missing data. A more complex analysis model will increase the complexity of any imputation model and the likelihood of imputation model misspecification.

## Supplementary Material

Supplementary Material

## Figures and Tables

**Table 1:** Missing data patterns of the times of onset of acute graft-vs-host disease (aGvHD) and relapse, according to the combination of events experienced, for 432 patients in the UK National Health Service Cord Blood Bank dataset. ✓ denotes observed time, x denotes missing time, and—denotes that the event was not experienced. Death times were completely observed.

Pattern	Event(s) experienced		Number of patients (%)
	aGvHD	Relapse	
1	✓	✓	36 (8%)
2	✓	✗	3 (1%)
3	✗	✗	5 (1%)
4	✓	-	145 (34%)
5	X	-	52 (12%)
6	-	✓	31 (7%)
7	-	✗	14 (3%)
8	-	-	146 (34%)

**Table 2:** Missing data mechanisms used in the simulation study for Scenarios 1-11.

Scenario	Probability of missing event times		
	Time to aGvHD	Time to relapse/death without aGvHD	Time to relapse/death after aGvHD
1. Times to aGvHD MAR	0.2 (1 + *z*_2*i*_)	0	0
2. Times to aGvHD MNAR (smallest times missing)	1 if *t*_*i*1_ < *t*_1(30%)_ 0 otherwise	0	0
3. Times to relapse/death MAR (conditional on aGvHD)	0	0.5 (1-0.8 *z*_2*i*_)	0
4. Time to relapse/death MAR (not conditional on aGvHD)	0	0.5 (1-0.8 *z*_2*i*_)	0.5 (1-0.8 *z*_2*i*_)
5. Time to relapse/death MNAR (smallest times missing)	0	1 if *t*_*i*2_ < *t*_RD(30%)_ 0 otherwise	1 if *t*_*i*3_ < *t*_RD(30%)_ 0 otherwise
6. Times to aGvHD MAR & times to relapse/death MAR	0.2 (1 + *z*_2*i*_)	0.5 (1-0.8 *z*_2*i*_)	0.5 (1-0.8 *z*_2*i*_)
7. Times to aGvHD MNAR (smallest times missing) and times to relapse/death MAR	1 if *t*_*i*1_ < *t*_1(30%)_ 0 otherwise	0.5 (1-0.8 *z*_2*i*_)	0.5 (1-0.8 *z*_2*i*_)
8. Times to aGvHD MNAR (largest times missing) and times to relapse/death MAR	1 if *t*_*i*1_ > *t*_1(70%)_ 0 otherwise	0.5 (1-0.8 *z*_2*i*_)	0.5 (1-0.8 *z*_2*i*_)
9. Times to aGvHD MAR and times to relapse/death MNAR (smallest times missing)	0.2 (1 + *z*_2*i*_)	1 if *t*_*i*2_ < *t*_RD(30%)_ 0 otherwise	1 if *t*_*i*3_ < *t*_RD(30%)_ 0 otherwise
10. Times to aGvHD times MAR and times to relapse/death MNAR (largest times missing)	0.2 (1 + *z*_2*i*_)	1 if *t*_*i*2_ > *t*_RD(70%)_ 0 otherwise	1 if *t*_*i*3_ > *t*_RD(70%)_ 0 otherwise
11. Times to aGvHD MNAR (smallest times missing) and times to relapse/death MNAR (smallest times missing)	1 if *t*_*i*1_ < *t*_1(30%)_ 0 otherwise	1 if *t*_*i*2_ < *t*_RD(30%)_ 0 otherwise	1 if *t*_*i*3_ < *t*_RD(30%)_ 0 otherwise

*Note*: In each scenario, for each patient *i, z*_2*i*_ = 1 for a double cord transplant and 0 otherwise; *t*_*ij*_ is the event time for patient *i* to the *j*^th^ state (*j* = 1: aGvHD, *j* = 2: relapse/death without aGvHD, *j* = 3: relapse/death after aGvHD); *t*_*j*(*p*%)_ is the *p*^th^ percentile of event times to the *j*^th^ state, ordered from smallest to largest; *t*_RD(*p*%)_ is the *p*^th^ percentile of all times to relapse/death (regardless of whether aGvHD was experienced or not), ordered from smallest to largest.

Abbreviations: aGvHD, acute graft-vs-host disease; MAR, missing at random; MNAR, missing not at random.

**Table 3:** Performance measures for estimates of the regression parameter γ_12_ in the transition intensity model from acute graft-vs-host disease (aGvHD) to relapse/death when some event times are MNAR.

Estimand			*γ*_12_			
Missing data mechanism	Multiple imputation method and analysis model		Bias	Mod SE	Std bias	Cov
MAR (aGvHD) and MNAR (smallest times to relapse/death)	PMMSUBGP, Cox		−0.001	0.004	−0.29	0.94
	PMMSUBGP, Weibull		<0.001	0.004	0.02	0.94
MAR (aGvHD) and MNAR (largest times to relapse/death)	PMMSUBGP, Cox		−0.001	0.005	−0.26	0.98
	PMMSUBGP, Weibull		0.007	0.005	1.44	0.66

*Note*: Parameter *γ*_12_ is for the time from transplant until aGvHD. PMMSUBGP, MI by Type 1 predictive mean matching with imputation models fit separately for patients with and without aGvHD.

Abbreviations: Cov, coverage; ModSE, average model-based SE; Std bias, standardized bias.

**Table 4:** Estimates and 95% confidence intervals (CI) of expected length of stay in each state in the first year post-transplant, comparing CCA and MI methods.

	Expected length of stay in each state (days)										
	CCA (N = 116)			PMM (N = 432)			PMMSUBGP (N = 432)			PMMSUBGP Weibull (N = 432)	
State	Est	95% CI		Est	95% CI		Est	95% CI		Est	95% CI
**Reference patient**											
Transplant	151	41-262		174	111-237		158	96-220		219	106-332
aGvHD	76	0-163		113	57-169		132	71-193		95	0-196
Relapse/death	123	10-237		70	23-117		68	24-112		51	0-130
**Low-risk patient**											
Transplant	273	136-365		229	152-306		233	160-306		291	212-365
aGvHD	38	0-116		95	25-165		94	25-163		54	0-123
Relapse/death	39	0-154		33	0-78		31	0-69		20	0-61
**High-risk patient**											
Transplant	33	0-179		72	0-171		139	27-251		165	0-335
aGvHD	9	0-89		22	0-55		49	0-99		36	0-93
Relapse/death	309	154-365		262	147-365		170	48-292		163	0-360

*Note*: 95% CI boundaries outside the range [0,365] were truncated to 0 and 365 for lower and upper bounds respectively. Unless otherwise stated, Cox transition intensity models were fitted. CCA, complete case analysis; PMM, MI by Type 1 predictive mean matching; PMMSUBGP, as for PMM with imputation models fit separately for patients experiencing (i) both aGvHD and cGvHD or cGvHD without aGvHD, (ii) aGvHD without cGvHD, (iii) relapse without a/cGvHD or neither a/cGvHD nor relapse. Reference patient has reference values of all categorical covariates and age 24 years. Low-risk patient has a non-malignant disorder, receives a high stem cell dose, age 4 years, with reference values of all other covariates. High-risk patient is in relapse at transplant, receives a double cord transplant, has 2 or more donor-recipient human leucocyte antigen mismatches (matching is important to avoid graft rejection), age 44 years, with reference values of all other covariates.

Abbreviations: aGvHD, acute graft-vs-host disease; cGvHD, chronic graft-vs-host disease.

## Data Availability

R code to perform the simulation study is provided in Supplementary Material, Section S5. The real data that support the findings of this study are not publicly available due to privacy restrictions.
